# Effects of Kisspeptin1 on Electrical Activity of an Extrahypothalamic Population of Gonadotropin-Releasing Hormone Neurons in Medaka (*Oryzias latipes*)

**DOI:** 10.1371/journal.pone.0037909

**Published:** 2012-05-23

**Authors:** Yali Zhao, Nancy L. Wayne

**Affiliations:** Department of Physiology, David Geffen School of Medicine at University of California Los Angeles, Los Angeles, California, United States of America; Georgia Health Sciences University, United States of America

## Abstract

Kisspeptin (product of the *kiss1* gene) is the most potent known activator of the hypothalamo-pituitary-gonadal axis. Both *kiss1* and the kisspeptin receptor are highly expressed in the hypothalamus of vertebrates, and low doses of kisspeptin have a robust and long-lasting stimulatory effect on the rate of action potential firing of hypophysiotropic gonadotropin releasing hormone-1 (GnRH1) neurons in mice. Fish have multiple populations of GnRH neurons distinguished by their location in the brain and the GnRH gene that they express. GnRH3 neurons located in the terminal nerve (TN) associated with the olfactory bulb are neuromodulatory and do not play a direct role in regulating pituitary-gonadal function. In medaka fish, the electrical activity of TN-GnRH3 neurons is modulated by visual cues from conspecifics, and is thought to act as a transmitter of information from the external environment to the central nervous system. TN-GnRH3 neurons also play a role in sexual motivation and arousal states, making them an important population of neurons to study for understanding coordination of complex behaviors. We investigated the role of kisspeptin in regulating electrical activity of TN-GnRH3 neurons in adult medaka. Using electrophysiology in an intact brain preparation, we show that a relatively brief treatment with 100 nM of kisspeptin had a long-lasting stimulatory effect on the electrical activity of an extrahypothalamic population of GnRH neurons. Dose-response analysis suggests a relatively narrow activational range of this neuropeptide. Further, blocking action potential firing with tetrodotoxin and blocking synaptic transmission with a low Ca^2+^/high Mg^2+^ solution inhibited the stimulatory action of kisspeptin on electrical activity, indicating that kisspeptin is acting indirectly through synaptic regulation to excite TN-GnRH3 neurons. Our findings provide a new perspective on kisspeptin's broader functions within the central nervous system, through its regulation of an extrahypothalamic population of GnRH neurons involved in multiple neuromodulatory functions.

## Introduction

Kisspeptin (a product of the *kiss1* gene) and its receptor (GPR54 or kiss1r) play major roles in the central regulation of the hypothalamo-pituitary-gonadal axis of vertebrates [1], [2], [3], [4]. In addition to its potent effects on hypophysiotropic GnRH neurons in control of reproduction, studies suggest that kisspeptin and its receptor play roles in regulating non-hypophysiotropic neurons, such as hippocampal dentate granule cells [5], hypothalamic anorexigenic proopiomelanocortin neurons and orexigenic neuropeptide Y cells [6] and serotonergic neurons in raphe nuclei [7]. Studies investigating central actions of kisspeptin on reproduction have focused primarily on mammalian species, showing that it has potent stimulatory effects on GnRH/gonadotropin secretion and electrophysiological activity of GnRH1 neurons in the hypothalamus and preoptic area (POA) [8], [9], [10], [11]. In mammals, *kiss1* expressing neurons are located in areas of the hypothalamus (anteroventral periventricular nucleus, periventricular nucleus, and arcuate nucleus) that ultimately regulate downstream gonadotropin secretion [1]. In rodents, kisspeptin has been shown to have both direct and indirect stimulatory actions on the electrical activity of GnRH1 neurons in the hypothalamus/POA [10], suggesting a complex neural network linking kisspeptin and GnRH neurons.

In teleost fishes, multiple forms of the kisspeptin gene (*kiss1* and *kiss2*) and kisspeptin receptor (kiss1r and kiss2r) have been identified in brain, including the species used in the present study – medaka (*Oryzias latipes*) [1], [12], [13], [14], [15]. *In situ* hybridization studies in medaka show that *kiss1* mRNA is located in hypothalamic nuclei (the nucleus posterioris periventricularis and the nucleus ventral tuberis) and ventromedial habenula; and, *kiss2* mRNA is located in the posterior tuberal nucleus and the periventricular hypothalamic nucleus [12], [13]. Importantly, biologically active peptide fragments of kisspeptin1 (kiss1) and kisspeptin2 (kiss2) have been shown to activate both kiss1r and kiss2r in fish [14], [15]. This suggests significant crosstalk between the different forms of kisspeptin and its receptors. However, studies in multiple species of fish have consistently shown that kiss2 is a more potent stimulator of central activation of gonadotropin secretion than kiss1 [13] [15]. This suggests that kiss2 is the primary regulator of the hypothalamo-pituitary-gonadal axis in fish, while kiss1 might act as a weaker modulator of the reproductive axis and have other physiological roles in the brain. On the other hand, recent work in medaka showed that kiss1 neurons, but not kiss2 neurons, are estrogen responsive and express estrogen receptor-alpha [16]. This latest finding suggests that kiss1 neurons are primarily involved in estrogen feedback and reproduction in medaka, throwing into question the relative roles of the different forms of kisspeptin in regulating reproduction.

Some teleost fishes have three distinct populations of GnRH neurons with different functions depending on their location. GnRH1 neurons located in the ventral telencephalon and POA are hypophysiotropic like their mammalian counterparts; GnRH2 neurons are located in the midbrain tegmentum; and GnRH3 neurons are located primarily in terminal nerve (TN) associated with the olfactory bulbs [2]. TN-GnRH3 neurons project widely throughout the brain (including to the olfactory bulbs, POA and hypothalamus), to the spinal cord, and retina, and are thought to play a neuromodulatory role in multiple systems, including olfactory, visual, and reproductive [17], [18], [19], [20], [21], and transmit information from the external environment to the central nervous system [22]. Thus, TN-GnRH3 neurons are considered important for coordinating multiple neural systems that regulate complex behaviors, including sexual motivation and arousal states [23]. In the present study, we hypothesized that kisspeptin1 regulates the physiology of extrahypothalamic TN-GnRH3 neurons in medaka. This would further our understanding of the broader functions for kisspeptin than its well-documented role in regulating the hypothalamo-pituitary-gonadal axis. Specifically, we investigated the effect of the biologically active 10-amino acid fragment of medaka kisspeptin1, kiss1(10), on electrical activity of TN-GnRH3 neurons using an intact brain preparation that preserves complex neural networks. We also investigated if kisspeptin's actions were due to direct effects on TN-GnRH3 neurons or through an indirect route involving synaptic transmission.

## Materials and Methods

### Animals

Medaka fish (*Oryzias latipes*) of the d-rR strain were used to generate a stable transgenic line in which the GnRH3 promoter drives expression of GFP in TN-GnRH3 neurons (gift of Dr. Kataaki Okubo, National Institute for Basic Biology, Okazaki, Japan) [24] [25]. Animals were maintained in an aquarium system on a 14L∶10D photoperiod at 28°C. They were fed twice daily with flake food and live brine shrimp. All procedures were carried out in accordance with and approved by the Animal Care and Use Committee of the University of California at Los Angeles.

### Experimental design

**Figure 1:**
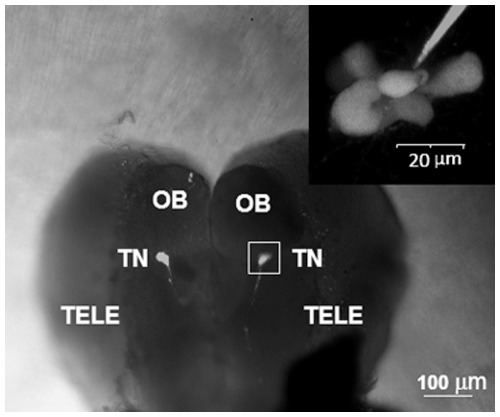
Ventral side of excised brain from adult transgenic medaka expressing GnRH3:GFP. (A) Anterior view of the brain showing the location of the olfactory bulbs (OB), bilateral clusters of GnRH:GFP neurons in the terminal nerve (TN), and telencephalon (TELE). (B) Higher magnification of the TN-GnRH3:GFP cluster in the box shown in panel A. In this example, the recording electrode was filled with fluorescent dye (Alexa Fluor 594, A-10442, Invitrogen, Carlsbad CA, USA) in order to better visualize the tip of the electrode relative to the neurons. All electrophysiological recordings were performed on GnRH3:GFP neurons in the TN clusters.

Adult male and female medaka were anesthetized by immersion in MS-222 (150 mg/L) and decapitated. The entire brain, from brainstem to olfactory bulb, was glued ventral-side up to a glass coverslip at the bottom of a flow-through recording chamber (P1; Warner Instrument Corp., volume of 0.75 ml). Temperature in the recording chamber was maintained at 21–22°C throughout the experiments (within the natural temperature range for this species). Following transfer to the recording chamber, the meninges covering the ventral side of the olfactory bulbs and telencephalon were peeled away, allowing access to the cells for electrophysiology. All recordings were performed on GnRH3:GFP expressing neurons located in the bilateral TN clusters of GnRH3 neurons associated with the olfactory bulb (Fig. 1). For all experiments, one neuron was recorded per brain.

The biologically-active, 10-amino acid fragment of medaka kiss1 (kiss1(10): YNLNSFGLRY-NH2) was synthesized by Bachem Inc. (Torrance, CA), based on the sequence described by Kanda and co-workers [12]. A control peptide was also synthesized (Bachem Inc.) in which the amino-acid sequence of kiss1(10) was randomized: NRYLYNFGSL-NH2. 800 µM stock solutions of kiss1(10) and the randomized 10-amino acid peptide were made in acidified albumin-saline (0.6 ml 12N HCl, 1.0 g Fraction V bovine serum albumin, 8.5 g NaCl per 1.0 L water; T. Pak, personal communication), stored at −80°C, and then diluted in fish saline just prior to experimentation. Solution (fish saline, described below) used during baseline and washout-recording periods was treated in the same way as the treatment solutions such that the final solutions throughout a given experiment contained the same amount of acidified albumin-saline.

A dose-response analysis was performed with seven kiss1(10) treatment groups (n = 5–11 neurons per group): 1 nM, 10 nM, 50 nM, 100 nM, 500 nM, 1000 nM, and 10,000 nM. An additional control group was treated with 100 nM of the randomized 10 amino-acid peptide described above. Neurons were recorded during baseline (at least 2 min), treatment (5 min), and washout (ranging from 5–30 min) periods. Because there was no sex difference in the rate of action potential firing during the baseline and treatment periods (data not shown), data from males and females were combined. In a second study, each neuron (n = 6) was recorded during a baseline period, followed by multiple doses of kiss1(10). Finally, to determine if kisspeptin is acting directly on TN-GnRH3 neurons or through an indirect mechanism, we tested the response of TN-GnRH3 neurons to 100 nM kiss1(10) by isolating synaptic inputs with bath application of a low Ca^2+^ (0.05 mM)/high Mg^2+^ (14 mM) fish saline [26] or by blocking action potentials with the sodium channel blocker tetrodotoxin (TTX; 0.5 µM) [25] (n = 7–8 neurons per group).

### Electrophysiology

Unless otherwise noted, chemicals were bought from Sigma-Aldrich, Inc. (St. Louis MO, USA). Loose-patch extracellular and whole-cell patch electrophysiological recordings of TN-GnRH3 neurons were carried out as previously described [25]. During recordings, aerated fish saline continuously bathed the brain and was perfused through the chamber at a rate of approximately 200 µl/min. Fish saline (3) contained 134 mM NaCl, 2.9 mM KCl, 2.1 mM CaCl_2_, and 1.2 mM MgCl_2_ in 10 mM Hepes. Low Ca^2+^/high Mg^2+^ fish saline containing 134 mM NaCl, 2.9 mM KCl, 0.05 mM CaCl_2_, and 14 mM MgCl_2_ in 10 mM Hepes was used to block synaptic transmission [26]. Osmolarity was adjusted to 290 mOsm with glucose, and pH was adjusted to 7.8 with NaOH. A solution containing 150 mM NaCl, 3.5 mM KCl, 2.5 mM CaCl_2_, 1.3 mM MgCl_2_, and 10 mM glucose in 10 mM Hepes was used for the loose-patch electrode [27]. Osmolarity was adjusted to 290 mOsm, and pH was adjusted to 7.4 with NaOH. A solution containing 112.5 mM potassium gluconate, 4 mM NaCl, 17.5 mM KCl, 0.5 mM CaCl2, 1 mM MgCl2, 5 mM MgATP, 1 mM EGTA, 10 mM Hepes, 1 mM GTP, 0.1 mM leupeptin, and 10 mM phosphocreatine was used for the whole-cell electrode. Osmolarity was adjusted to 290 mOsm by titrating the final volume of water, and pH was adjusted to 7.2 with KOH.

TN-GnRH3 neurons expressing GFP were visualized under an upright microscope (BX50W, Olympus, Melville, NY, USA) using a combination of infrared differential-contrast (IR-DIC) optics and an IR-camera (OL-1500, Olympus), and in the presence of both UV- and brightfield illumination. Electrical activity was monitored either with a loose-patch electrode (resistance 6–10 M Ohms) or a whole-cell patch electrode (resistance 7–10 M Ohms) pulled from borosilicate glass (1.5 mm diameter, WPI, Sarasota, FL; P87, Sutter Instruments, Novato, CA, USA). For loose-patch recording, a low-resistance seal (30–100 M Ohms) was obtained following release from positive pressure. For whole-cell recordings, after forming a high-resistance seal (>3 G Ohms) by applying negative pressure, a second pulse of negative pressure was used to rupture the membrane. Data were collected if series resistance was less than 35 M Ohms and if interspike membrane potential (IS Vm) was at least - 40 mV under control (baseline) conditions. Recordings of action potentials and Vm were obtained using an Axopatch 200B amplifier (Axon Instruments, Foster City, CA, USA) in current-clamp mode, and digitized with an ITC-18 computer interface (Instrutech Corp., Port Washington, NY, USA). Recordings were monitored online using both AxoGraph software (Axon Instruments, Foster City, CA, USA) and PowerLab data acquisition and analysis instrumentation and software (ADInstruments Inc., Colorado Springs, CO, USA), and stored off-line for subsequent AxoGraph data analysis of spike frequency and Vm. Data were collected once the baseline recording stabilized (usually within 5 min from seal formation).

### Data analysis

Values are shown as the mean ± standard error of the mean. Frequency of action potential firing was analyzed in one-minute bins of recording during baseline, treatment, and washout periods. In experiments with three or more groups, changes in the frequency of action potential firing in response to treatment were analyzed by either one-way analysis of variance (ANOVA) or repeated measures ANOVA, followed by Newman-Keul's multiple comparison test (Prism, GraphPad Software, Inc., La Jolla, CA). In the experiments with two groups, Vm and interspike Vm were analyzed by two-tailed paired t-test. Values were considered significantly different if P<0.05.

## Results

**Figure 2:**
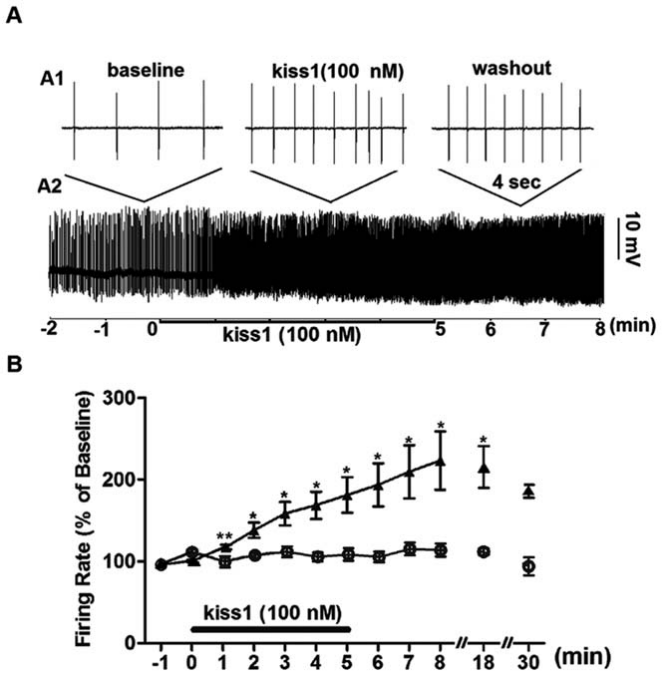
Effect of 100 nM kiss1(10) on spontaneous action potential firing of TN-GnRH3:GFP neurons. (A) Ten-min continuous loose-patch recording trace from a representative neuron showing 2 min of the baseline period, followed by five min of 100 nM kiss1(10) treatment, followed by a washout period. A2 shows the continuous recording; A1 shows expanded four-second excerpts from the baseline, treatment, and washout periods. (B) Summary of spike frequency normalized to baseline (% of baseline). The experimental group (triangle symbols; 11 neurons from 11 fish, up through 10 min of recording) was treated with 100 nM kiss1(10) for 5 min; the control group (circle symbols; 4 neurons from 4 fish) was treated with baseline solution. Data from continuous recordings were counted in one-minute consecutive bins, and compared to baseline. In the experimental group, the washout period was of variable duration (5–30 min) depending on how long a stable recording could be held. The final two time points from the experimental group show normalized spike frequency at the 13^th^ (n = 6 neurons) and 25^th^ (n = 2 neurons; not statistically analyzed) minute after start of washout (18 and 30 min after start of kiss1 treatment, respectively). In the control group, all 4 neurons were recorded successfully for 30 min, and there was no statistically significant change in spike frequency compared to baseline. Asterisks denote statistically significant differences between treatment/washout periods and baseline. *: P<0.01; **: P<0.001, one-way ANOVA followed by Neuman-Keuls multiple comparison test.

**Figure 3:**
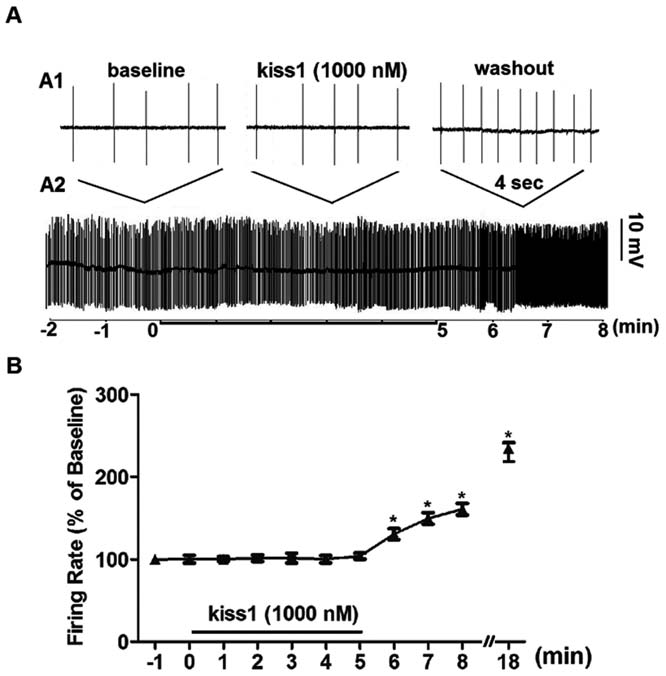
Effect of 1000 nM kiss1(10) on spontaneous action potential firing of TN-GnRH3:GFP neurons. (A) Ten-min continuous loose-patch recording trace from a representative neuron showing 2 min of the baseline period, followed by five min of 1000 nM kiss1(10) treatment, followed by a washout period. A2 shows the continuous recording; A1 shows expanded four-second excerpts from the baseline, treatment, and washout periods. (B) Summary of spike frequency normalized to baseline (% of baseline) from 7 neurons from 7 fish (up through 10 min of recording). Data from continuous recordings were counted in one-minute consecutive bins, and compared to baseline. The washout period was of variable duration (5–13 min) depending on how long a stable recording could be held. The final time point shows normalized spike frequency at the 13^th^ minute after start of washout (18 min after start of kiss1 treatment; n = 4 neurons). Asterisks denote statistically significant differences between treatment/washout periods and baseline. *: P<0.01, one-way ANOVA followed by Neuman-Keuls multiple comparison test.

**Figure 4:**
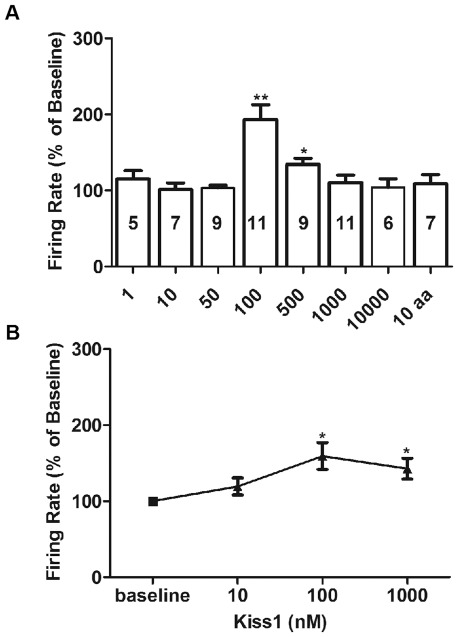
Dose-response analysis of kiss1(10) on the rate of action potential firing from TN-GnRH3 neurons. (A) Loose-patch recording data are shown as the spike frequency normalized to baseline (% of baseline) for kiss1(1) treatments ranging from 1–10,000 nM. Each treatment dose represents data from a different group of fish; numbers inside the histogram bars indicate number of neurons recorded. A control group (10 aa) was treated with 100 nM of a synthetic peptide in which the 10 amino-acid sequence of kiss1(10) was randomized. Asterisks denote statistically significant differences between treatment and baseline. *: P<0.05; **: P<0.001, one-way ANOVA followed by Neuman-Keuls multiple comparison test. Further analysis showed that the electrophysiological response to the 500 nM dose was significantly lower than to the 100 nM dose (P<0.05). (B) Comparison of spike frequency (% of baseline) in response to consecutively increasing doses (10 nM, 100 nM, 1000 nM) of kiss1(10) for each neuron (n = 6 neurons). Asterisks denote statistically significant differences between treatment and baseline. *: P<0.05, one-way repeated measures ANOVA followed by Neuman-Keuls multiple comparison test. Further analysis showed no significant difference in spike frequency between the 100 nM and 1000 nM treatment periods. In both A and B, data were analyzed from the last 1-min of baseline recording and compared to that from the last 1-min of kiss1(10) treatment.

Figures 2,3,4 show the effect of different doses of kiss1(10) on the rate of action potential firing of TN-GnRH3 neurons from the intact brain of adult medaka, as determined by loose-patch electrophysiology. Treatment with 100 nM kiss1(10) had a rapid (within 60 sec) and robust stimulatory effect on spike frequency (89% increase over baseline at the end of the 5-min treatment period) (Fig. 2). The stimulatory response was long lasting and was not eliminated during the washout period (up to 30 min after the start of washout), similar to what was reported with murine hypothalamic/POA GnRH1 neurons [8] [10]. A control group was included in this study in which neurons were continuously treated with 0 nM kiss1(10) (‘baseline’ saline solution) for 30 min, with no significant change in spike frequency. This indicates that the prolonged increase in electrical excitation in the experimental group was due to long-lasting stimulatory actions of kiss1(10), and not due to deterioration of the recording conditions leading to non-specific membrane depolarization. Surprisingly, treatment with 1000 nM kiss1(10) had no significant effect on spike frequency, until during the washout period when the concentration of kiss1(10) would have declined (Fig. 3). Reversal of this non-responsiveness during treatment was very rapid, occurring within 30 sec of when the washout solution reached the preparation bath. Notably, unlike the washout response, there was no transient increase in spike frequency as the concentration of kiss1(10) built up towards 1000 nM during the wash-in period. We have no explanation for this asymmetrical response.

Fig. 4A shows a dose-response analysis of kiss1(10) on spike frequency from different groups of TN-GnRH3 neurons. Data from the final minute of the treatment periods were analyzed and then normalized to the final minute of the baseline periods for each neuron. Unlike with the 100 nM dose, treatment with 1, 10, and 50 nM kiss1(10) had no significant effect on spike frequency compared to the baseline period. Likewise, treatment with 100 nM of the randomized sequence of kiss1(10) amino acids had no effect on spike frequency – which is very different from the stimulatory effect seen when preparations were treated with the same dose of active kiss1(10) peptide. This indicates that the stimulatory effect of 100 nM kiss1(10) is specific, and not due to undefined substances in the treatment solution. Treatment with 500 nM kiss1(10) had a significant stimulatory effect on spike frequency (34% increase over baseline), but it was less robust than that seen with the lower dose of 100 nM (100 nM vs 500 nM, P<0.05). As shown in Fig. 3, the higher dose of 1000 nM did not stimulate the rate of action potential firing; similarly, the highest dose of 10,000 nM kiss1(10) did not stimulate nor inhibit spike frequency. To determine if the lack of response to 1000 nM kiss1(10) was due to desensitization of the TN-GnRH3 neurons to high concentrations of the neuropeptide, each neuron was recorded during baseline and then treated with successive increasing doses of kiss1(10). Six neurons were successfully recorded during baseline and 5-min consecutive treatment periods with three concentrations of kiss1(10): 10 nM, 100 nM, and 1000 nM (Fig. 4B). If the non-responsiveness seen in Fig. 3 was due to desensitization, then treatment with 1000 nM kiss1(10) might inhibit the excitatory response elicited by treatment with 100 nM of the neuropeptide. Once again, the 100 nM dose significantly stimulated spike frequency compared to baseline. This elevated spike frequency continued during treatment with 1000 nM kiss1(10) and was not significantly altered compared to treatment with 100 nM kiss1(10), indicating no inhibitory effect with the higher dose of neuropeptide.

**Figure 5:**
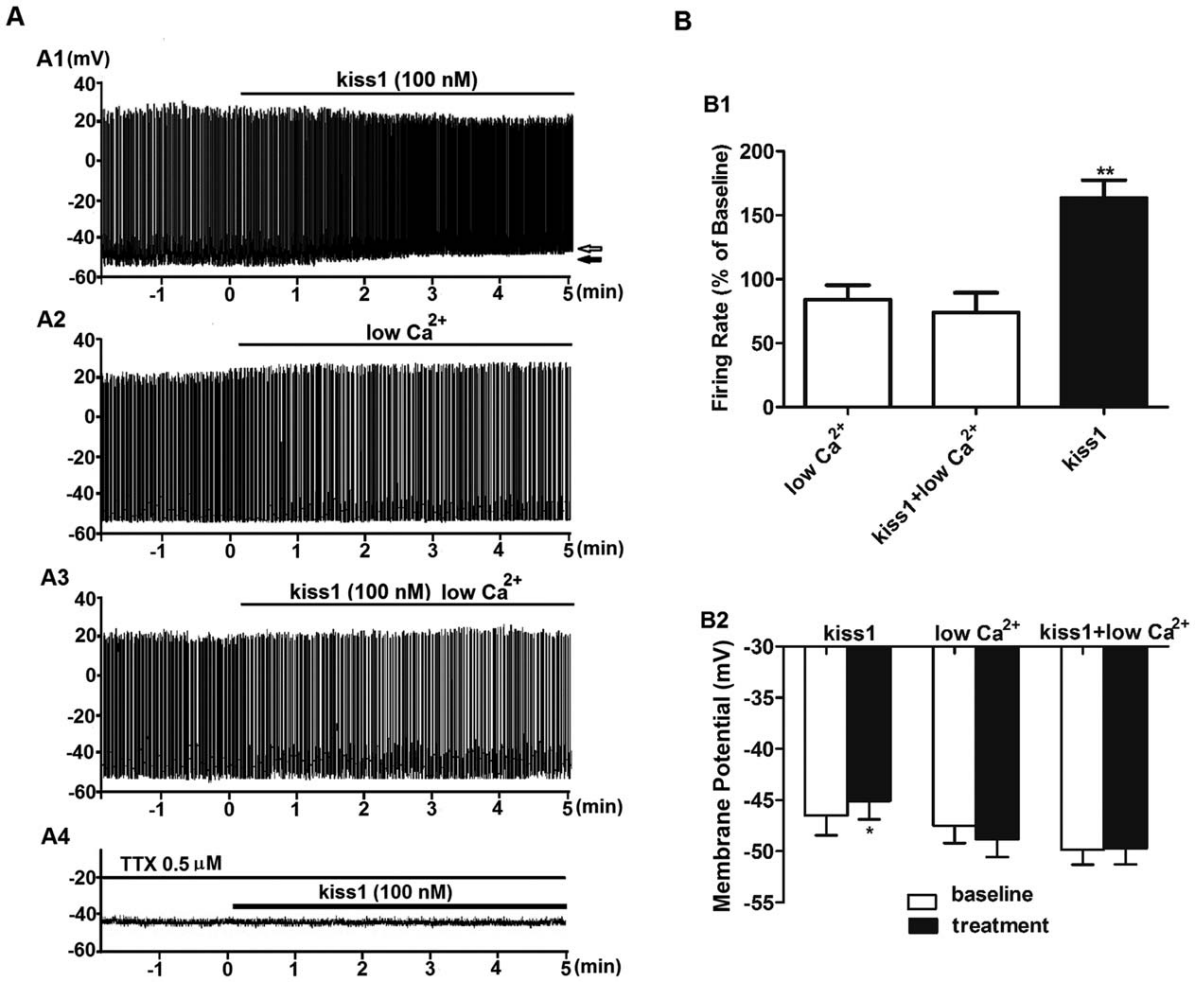
Kisspeptin stimulates TN-GnRH3 neurons through an indirect effect involving synaptic transmission. (A) Representative traces from whole-cell patch electrophysiology recordings. A1: 100 nM Kiss1(10) treatment leads to slight depolarization of membrane potential (Vm), accompanied by increased spike frequency. At the right of the trace: the black arrow shows the interspike (IS) Vm (−47 mV) during the last minute of recording from the baseline period, and the open arrows shows the IS Vm (−45.2 mV) during the last minute of recording from the kiss1(10)-treatment period. A2: Low Ca^2+^/high Mg^2+^ saline alone. A3: 100 nM Kiss1(10) in low Ca^2+^/high Mg^2+^ saline.A4: 100 nM Kiss1(10) in 0.5 µM TTX. (B) Summary and analysis of the data. B1: Comparison of the firing rate (% of baseline) of TN-GnRH3 neurons. Low Ca^2+^/high Mg^2+^ blocks the kiss1(10) induced increase of action potential firing frequency. B2: Vm or IS Vm of pretreatment conditions (white histograms) (baseline normal saline or TTX, as indicated) compared to four different treatments (black histograms): 100 nM kiss1(10); low Ca^2+^/high Mg^2+^; 100 nM kiss1(10) in the presence of low Ca^2+^/high Mg^2+^; 100 nM kiss1(10) in the presence of TTX. * indicates P<0.05; ** indicates P<0.01; paired t-test. In all cases, Vm and IS Vm data were analyzed from the final one min of pretreatment and treatment periods.

To determine if kiss1(10) is acting directly or indirectly on TN-GnRH3 neurons to stimulate their electrical activity, we analyzed the neurons' response to 100 nM kiss1(10) using whole-cell patch electrophysiology recording after blocking action potential firing with TTX (0.5 µM) and blocking synaptic transmission with low Ca^2+^/high Mg^2+^ solution [25], [26]. Whole-cell patch electrophysiology revealed that 100 nM kiss1(10) treatment caused a small, but significant depolarization of interspike Vm (Fig. 5, A1 and B2) – enough to stimulate an increase in spike frequency (Fig. 5, A1 and B1) [28]. Use of TTX to block action potential firing led to significant membrane depolarization (Baseline control: 50.44±1.9 mV, n = 7; TTX: 40.14±1.88 mV, n = 8, P<0.01)), which has been observed in other neuronal systems [29]. Following treatment with TTX [25], kiss1(10) did not cause further membrane depolarization (Fig. 5, A4 and B2). Because it is possible that TTX-induced depolarization masked excitatory actions of kiss1(10), a second approach was used to clarify if kiss1(10) excites TN-GnRH3 neurons through regulating synaptic inputs. In this additional study, TN-GnRH3 neuron electrical activity was recorded in the presence or absence of 100 nM kiss1(10) together with a low Ca^2+^/high Mg^2+^ solution to block synaptic transmission (n = 7 neurons/group) [26]. 100 nM kiss1(10) treatment in low Ca^2+^/high Mg^2+^ solution had no effect on the rate of action potential firing or interspike Vm (Fig. 5, A3, B1, and B2). These results suggest that kisspeptin stimulates electrical activity of TN-GnRH3 neurons indirectly by regulating synaptic inputs.

## Discussion

A growing body of studies shows that kisspeptin plays important roles in control of puberty and reproduction by strongly stimulating hypophysiotropic GnRH neurons located in the hypothalamus and preoptic area [8], [30]. Kisspeptin stimulates GnRH neurons either directly through activation of kiss1r on GnRH neurons or indirectly by regulating synaptic inputs via kiss1r on interneurons [6], [8], [10]. Taking advantage of medaka with multiple populations of GnRH neurons with different functions [2], this is the first study demonstrating kisspeptin's actions on an extrahypothalamic population of GnRH neurons in any model system, as well as the first report of kisspeptin modulating electrical activity of GnRH neurons in fish. Given the importance of TN-GnRH3 neurons in modulating the activity of a broad spectrum of neural regions and its role in sensory information processing [23], the present findings provide important information in our understanding of kisspeptin's broad role in brain functions. We found that TN-GnRH3 neurons were sensitive to the stimulatory actions of kiss1(10), with a 100 nM dose having a robust effect on frequency of action potential firing. Further, kiss1(10) had long-lasting actions on TN-GnRH3 neuron activity – even many minutes after terminating treatment, spike frequency was still significantly elevated above pretreatment levels. This robust and long-lasting electrophysiological response in the medaka model system is very similar to what was seen in murine brain-slice preparations containing hypophysiotropic GnRH1 neurons treated with mammalian kisspeptin [8], [10].

The present study showed that kiss1(10) stimulated TN-GnRH3 neurons in a narrow dose range (100–500 nM), while relatively high doses kiss1(10) (1000 and 10,000 nM) were ineffective in altering the electrical activity of TN-GnRH3 neurons – neither stimulating nor inhibiting the frequency of action potential firing. And although decreasing the dose to 500 nM stimulated spike frequency above baseline, it was significantly less stimulatory than the lower dose of 100 nM – suggesting a fairly narrow activational range of kisspeptin1 on the electrical activity of TN-GnRH3 neurons. Previous studies in rats and non-human primates showed that either continuous treatment with kisspeptin or high doses of the peptide led to inhibition of the reproductive axis. Both acute treatment with high doses and chronic treatment with low doses of kisspeptin caused testicular degeneration in rats through central mechanisms [31], [32]. Work in male rhesus monkeys showed that continuous treatment with kisspeptin inhibited LH secretion via desensitization of kisspeptin receptors [33]. Presumably, this type of desensitization observed in earlier studies occurred through lack of stimulation or inhibition of GnRH neuron activity. In the present study, the non-responsiveness of TN-GnRH3 neurons with the higher dose of 1000 nM kiss1(10) in two separate experiments does not support a classic desensitization mechanism at work in the medaka model system. A clinical study in men revealed intravenous kisspeptin-10 boluses evoked rapid and potent LH secretion within a narrow dose range, with no significant increase of LH after administering the highest dose [34]. Similarly, high doses of kisspeptin treatment had no effect on the activation of *c-fos* in zebrafish habenula, whereas lower doses were excitatory [7]. One possible explanation for this phenomenon is that high concentrations of kisspeptin-10 may stimulate another RF-amide receptor (gonadotropin inhibitory hormone receptor, GnIH-r), which has inhibitory effects on GnRH and LH secretion, and could cancel out the stimulatory effects that it has on activating kiss1r [34]. Notably, increased firing rate in response to a single dose of 100 nM kiss1(10) appeared greater than the firing rate in response to the same dose of kiss1(10) when it is part of an increasing series of dose applications (see Fig. 4), suggesting that pretreatment with a low concentration of kisspeptin (below stimulating threshold) could partly desensitize the receptors thereby reducing the response to kiss1(10). However, previous work using a luceriferase reporter assay of fish kisspeptin receptor activation showed that 1000 nM kisspeptin did not cause receptor desensitization [14], [15]. Those studies support the idea that non-responsiveness of TN-GnRH3 to relatively high doses of kiss1(10) in the present experiment was not due to classic receptor desensitization.

Whole-cell electrophysiology recording revealed that the stimulatory effect of kiss1(10) on the frequency of action potential firing was due to its causing a small, but significant, depolarization of Vm. Previous work in medaka TN-GnRH3 neurons showed that a 5–7 mV depolarization significantly inhibited both the amplitude of action potentials and spike frequency due to Na^+^ channel inactivation [28]. Because TN-GnRH3 neurons are relatively depolarized under normal conditions [19], [25], [28], further depolarization can push Na^+^ channels into an inactivated state. In the present study, the slight depolarization of 1.6 mV in response to kiss1(10) was associated with increased spike frequency; further depolarization would most likely inactivate Na^+^ channels and inhibit the neurons' electrical activity [28].

Previous work showed that kisspeptin has both direct and indirect actions on murine hypothalamic GnRH1 neurons to stimulate spike frequency [10]. The direct effect is achieved by regulating multiple ion channels through a phospholipase C/calcium–dependent pathway via activation of kiss1r on GnRH neurons [35]. A direct action is corroborated by work in cichlid fish showing that GnRH1, GnRH2, and GnRH3 neurons express the kisspeptin receptor [36]; however, a similar study has not yet been reported for medaka in which responses to kisspeptin treatment differs from that in other fishes. In the present study, after blocking action potential firing with TTX, kiss1(10) didn't induce further membrane depolarization, suggesting that kisspeptin does not act on TN-GnRH3 neurons directly. However, prolonged treatment of the whole brain with 0.5 µM TTX depolarized TN-GnRH3 neurons by about 10 mV, which may have been caused by the inactivation of both the TTX-sensitive sodium channel and the TTX-resistant sodium channel [29], [37]. This depolarization may have masked possible kiss1-induced depolarization, and so an additional experiment was performed. To test if kisspeptin excites TN-GnRH3 neurons indirectly through affecting afferent synaptic inputs, we blocked synaptic transmission with application of a low Ca2+/high Mg2+ solution [25]. We found that blocking synaptic transmission abolished the kiss1(10) stimulatory effects on TN-GnRH3 neurons; no increase of neuron activation and membrane depolarization were observed, indicating that kisspeptin affects TN-GnRH3 neuron activity indirectly through synaptic regulation of neural circuits. This supports the observations noted by Mitani and co-workers [16] that there is considerable heterogeneity in the GnRH-kisspeptin network between species, including different species of teleost fishes.
